# Pilot study comparing sleep logs to a commercial wearable device in describing the sleep patterns of physicians-in-training

**DOI:** 10.1371/journal.pone.0305881

**Published:** 2024-07-22

**Authors:** Amanda B. Hassinger, Misol Kwon, Jia Wang, Archana Mishra, Gregory E. Wilding

**Affiliations:** 1 Department of Pediatrics, Division of Pulmonology and Sleep Medicine, University at Buffalo School of Medicine and Biomedical Sciences, Buffalo, New York, United States of America; 2 Attending Physician, John R. Oishei Children’s Hospital, Buffalo, New York, United States of America; 3 Division of Sleep Medicine, University of Pennsylvania Perelman School of Medicine, Philadelphia, Pennsylvania, United States of America; 4 University of Buffalo School of Nursing, Buffalo, New York, United States of America; 5 Department of Biostatistics, University at Buffalo School of Public Health and Health Professions, Buffalo, New York, United States of America; 6 Department of Medicine, Division of Pulmonology, University at Buffalo Jacobs School of Medicine and Biomedical Sciences, Critical Care and Sleep Medicine, Buffalo, New York, United States of America; Helwan University Faculty of Engineering, EGYPT

## Abstract

With the increasing burden of professional burnout in physicians, attention is being paid to optimizing sleep health, starting in training. The multiple dimensions of physicians’ sleep are not well described due to obstacles to easily and reliably measuring sleep. This pilot study tested the feasibility of using commercial wearable devices and completing manual sleep logs to describe sleep patterns of medical students and residents. Prospective pilot study of 50 resident physicians and medical students during a single year of training. Participants completed a manual sleep log while concurrently wearing the Fitbit Inspire device for 14-consecutive days over three clinical rotations of varying work schedules: light, medium, and heavy clinical rotations. Study completion was achieved in 24/50 (48%) participants. Overall correlation coefficients between the sleep log and Fitbit were statistically low; however, the discrepancies were acceptable, i.e., Fitbit underestimated time in bed and total sleep time by 4.3 and 2.7 minutes, respectively. Sleep onset time and waketime were within 8 minutes, with good agreement. Treatment of sleep episodes during the day led to variance in the data. Average missingness of collected data did not vary between medical students or residents or by rotation type. When comparing the light to heavy rotations, hours slept went from 7.7 (±0.64) to 6.7 (±0.88), quality-of-life and sleep health decreased and stress, burnout, and medical errors increased. Burnout was significantly associated with worse sleep health, hours worked, and quality-of-life. Prospective data collection of sleep patterns using both sleep logs and commercial wearable devices is burdensome for physicians-in-training. Using commercial wearable devices may increase study success as long as attention is paid to daytime sleep. In future studies investigating the sleep of physicians, the timing of data collection should account for rotation type.

## Introduction

Professional burnout is an epidemic affecting a large proportion of healthcare providers, as early as the first year of medical school [1]. Data show 50% of medical students [2], 56% of interns [3], 40% of practicing surgeons [4], and 46% of all attending physicians across medicine sub-specialties [5] experience burnout which may result from emotional exhaustion and high workload leading to inadequate and irregular sleep. The consequences of burnout include committing medical errors, depression, suicide, substance abuse, and is suspected to be driving a physician shortage [2,6–11].

One of the modifiable risk factors consistently associated with burnout and physician well-being is sleep [12,13]. Although sleep duration or total number of hours slept is the most often described metric, evidence is emerging that sleep health has multiple dimensions that describe consistent sleep at the correct time without interruption. Work hour rules have forced healthcare workers into shorter shifts abruptly changing from nighttime to daytime, often against the natural circadian rhythm. These types of abrupt shift changes have been documented to cause depression, burnout, cognitive deficits, and motor vehicle accidents among emergency room physicians [14]. However, their impact on physicians-in-training is not thoroughly explored.

Gaining insight into the sleep health of residents and medical students is hindered by the difficulty of characterizing the granular sleep patterns in this actively overworked group. Research-grade actigraphy devices are the gold standard for measuring sleep duration, timing, efficiency, and regularity in outpatient settings; however, they are expensive and require specialized software and expertise to analyze and interpret the data. On the other hand, there are affordable and user-centric commercial wearable devices that offer a modest amount of sleep data that can be transmitted through cloud systems, eliminating the requirement for proprietary software. For this reason, there is a growing volume of studies employing such commercial devices, for example, in research involving surgery residents [15], and internal medicine residents [16]. Nevertheless, in these investigations, there is still a lack of comparisons with manual sleep logs to ascertain the variance between self-report and sleep data derived from commercial wearable devices.

This pilot study compared manual sleep logs to commercial wearable devices in medical students and residents to determine measurement discrepancy to inform feasibility and design of future large-scale studies. The primary aim of this study was to evaluate the correlation between sleep variables (i.e., time in bed [TIB], total sleep time [TST], sleep efficiency [SE%], sleep onset time, and waketime) reported on manual sleep log and that measured by the Fitbit Inspire.^TM^ Our hypothesis was that there would be insignificant differences in TIB, TST, SE%, sleep onset time, and waketime collected via Fitbit when compared to the manual sleep log. Second, we sought to determine study design viability for physicians-in-training to complete the entire protocol.

## Materials and methods

This prospective observational trial enrolled 25 medical students and 25 resident physicians over a single year of training from July 1, 2020 to June 30, 2021. At enrollment, participants were given a Fitbit Inspire^TM^ and directed to complete a baseline survey. Instructions were then provided on how to register the Fitbit device and synch it with a de-identified cloud platform which was developed for research purposes only. Study participation involved completing a manual sleep log (i.e., paper diary or on a personalized online link to a secure web-based data collection tool [REDCap], based on participant preference) for 14-consecutive days while wearing the Fitbit at three timepoints over the one year of training. For ethical reasons, only the dates entered in the manual sleep log were extracted from the Fitbit database.

Participants were instructed on selecting the rotations during which they should wear the device. The rotations were described as: (1) *a light rotation* with no nights or weekend shifts and primarily outpatient work, such as an elective or clinic-based rotation, (2) *a medium rotation* that involved clinical time in a hospital but no night shifts or weekends, (3) *a heavy rotation* that involved night and weekend shifts, such as in the Emergency Department or Intensive Care Unit.

In addition to entering their sleep patterns on sleep logs for 14-consecutive days on each rotation, participants were also asked to provide the following via REDCap survey: number of days off over the 4-week rotation, missed significant life experiences, commission of a medical error, health-related quality-of-life score, the abridged Maslach Burnout Scale [17], 2-items from the Patient Health Questionnaire related to depression [18], and a 10-item Perceived Stress Scale (PSS) [19]. PSS score above 12 indicated moderate to severe stress, as has been previously reported [19]. Frequency of habits known to impact sleep (i.e., screen time, alcohol, caffeine, and tobacco use before bed) were also elicited.

A composite sleep health score was calculated using 6 domains: *regularity* (i.e., “How often do you have a set sleep schedule?”), *duration* (i.e., “How often do you get 6 to 8 hours of sleep per day?”), *efficiency* (i.e., “How often do you spend less than 30 minutes awake total while trying to sleep?”), *timing* (i.e., “How often are you asleep between 2 and 4 am?”), *quality* (i.e., “How often do you wake up refreshed?”) and *daytime sleepiness* (i.e., “How often do you stay awake all day without napping or dozing off?”) [20]. A six-point Likert scale quantified frequency of each domain with higher number indicative of healthier sleep patterns. This sleep health score has been validated in large population studies and been shown to correlate well with other measures of sleep as well as daytime health [21–26].

At the time of study completion or exit, all participants were prompted to complete an Exit Questionnaire with 5 questions about the feasibility of completing all the required tasks for the study (i.e., reasons for not completing, length taken to complete, other recommendations, etc.). Monetary compensation was provided to participants who completed the baseline survey, sleep logs, surveys and wearing the Fitbit device at all three rotation time points, and the Exit Questionnaire.

The study was approved after full review by the Institutional Review Board of the University at Buffalo. All participants provided written informed consent.

### Statistical analysis

Descriptive statistics were used to summarize the sample and inferential statistics to compare the sleep and rotational data between each rotation type. Regression was used to correlate burnout scores, stress, quality-of-life, hours worked, and hours slept. Bland-Altman plots and concordance correlation coefficients (CCC) for agreement were used to assess the agreement between sleep variables obtained from the sleep log and from the Fitbit device for each individual subject [27]. The strength of the association between the two methods of obtaining sleep variables was measured with Pearson correlation coefficients.

We additionally examined the relationship between the data input missingness of the sleep log and both rotation type and training level (i.e., medical student or resident). Descriptive statistics were computed to examine the distribution of the missingness of each rotation type. The comparison of missingness between different rotation types was performed by Kruskal-Wallis rank sum test. Due to the hierarchical nature of the data (i.e., observations nested within participants), the assumption of independence of observations was violated and thus, multilevel modelling was used. Linear mixed effects model was used to investigate different independent variables’ effect on missingness. Statistical analyses were performed using R software version 4.2.2 and SPSS, version 29.

## Results

The description of the enrolled participants is displayed in Table 1. Of the 25 medical students recruited, 12 were 3^rd^ years and 13 were 4^th^ year medical students. Residents were interns (9, 36%), 2^nd^ years (9, 36%) or 3^rd^ years (7, 28%) in Internal Medicine (11, 44%), Pediatrics (11, 44%) or Medicine-Pediatric (3, 12%) programs. The majority were female (62%), white (61%), non-Hispanic (90%), and were in a relationship (70.8%). Compared to medical students, residents more often had their origins in different geographic regions or countries other than US, with Canada and various Asian countries being the most common.

**Table 1:** Description of the physicians-in-training who participated in a pilot study in 2020–2021 investigating the feasibility of manual sleep logs and commercial wearable devices to describe sleep patterns.

	All Participants (n = 50)	Medical Students (n = 25)	Residents (n = 25)	p-value
Age (years)	26.9 (2.4)	25.6 (1.8)	28.3 (2.1)	<0.001
Body mass index (kg/m^2^)	23.8 (3.3)	24.2 (3.6)	23.5 (2.9)	0.456
Female sex, n(%)	31 (62%)	17 (68%)	14 (56%)	0.382
Non-Hispanic, n(%)	44 (88%)	23 (92%)	21 (84%)	0.585
White, n(%)	30 (60%)	18 (72%)	12 (48%)	0.075
Works/trains in their region of origin	19 (38%)	16 (64%)	3 (12%)	<0.001
Non-United States citizen	16 (32%)	1 (4%)	15 (60%)	0.005
Hours to travel to get home	2 (0–8)	0 (0–5)	8 (1–19)	<0.001
In a serious committed relationship	34 (68%)	17 (68%)	17 (68%)	1.000
History of insomnia	4 (8%)	2 (8%)	2 (8%)	1.000
History of obstructive sleep apnea	1 (2%)	0	1 (4%)	1.000
History of anxiety or depression	5 (10%)	3 (12%)	2 (8%)	1.000

Variables are presented as mean (SD), median (IQR1-3) or n (proportion of the column) unless otherwise specified. P-values were obtained though t-tests for the continuous variables or Chi-square or Fischer exact tests for the categorical ones.

### Comparison between manual sleep log and Fitbit values

**Fig 1:**
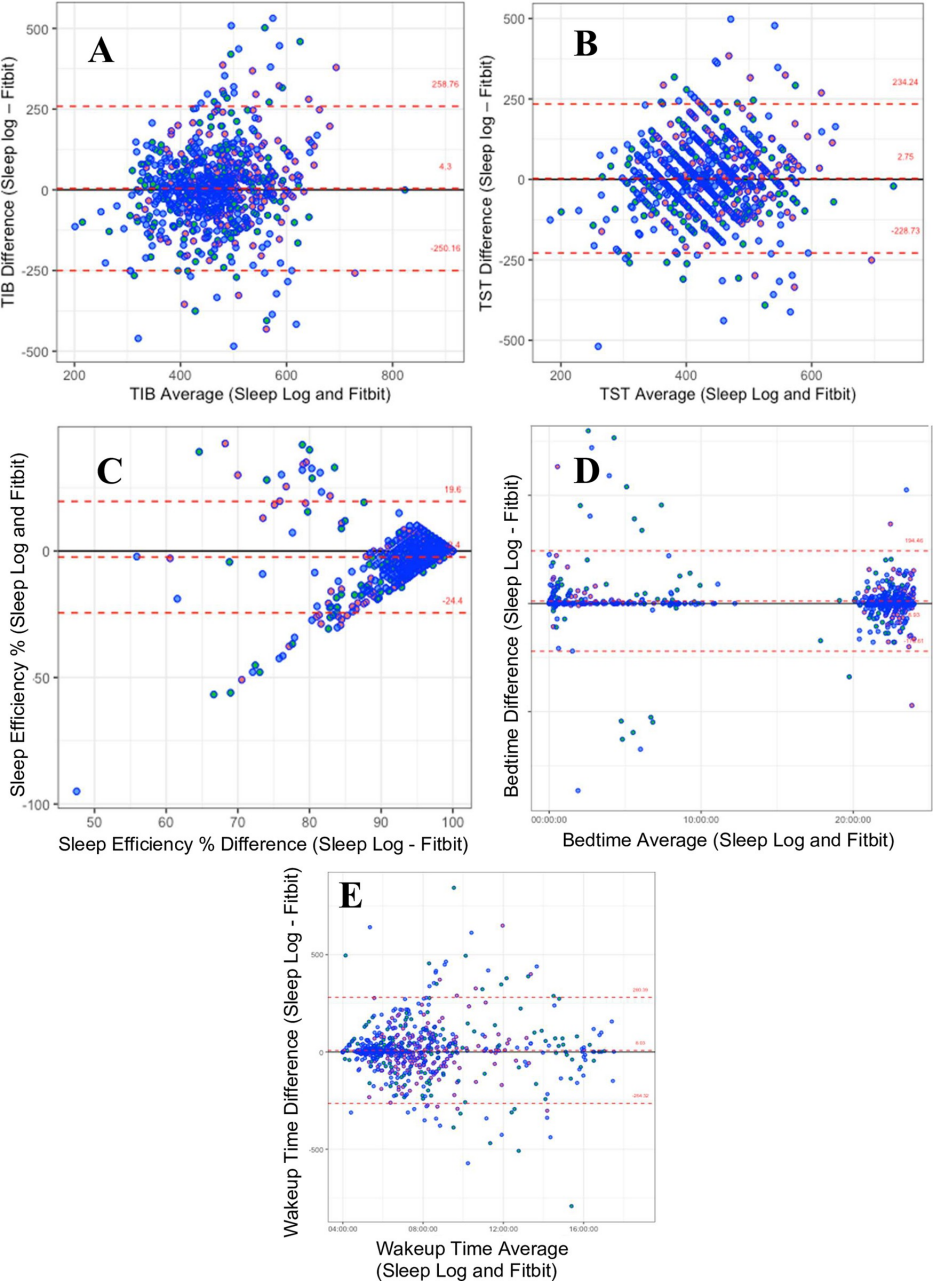
A-E. Bland-Altman plots comparing sleep data from FitBit to sleep logs. Plots compare (A) time in bed (TIB), (B) total sleep time (TST), (C) sleep efficiency (SE%), (D) sleep onset time, and (E) waketime as collected from sleep logs and the Fitbit Inspire device in 50 resident physicians and medical students. The data-points in red are from the light rotation, green from the medium and blue from the heavy clinical rotation. Mean difference ± 1.96 standard deviations (SD) are displayed for each Bland-Altman plot.

There was a total of 1,446 paired sleep log entries (n = 723) and Fitbit sleep episodes (n = 723) available for direct comparison of TIB, TST, SE% (i.e., TST/TIB x 100%), sleep onset time, and waketime. Table 2 shows the comparisons between the sleep variables obtained from the manual logs as compared to the Fitbit device. While the absolute mean differences for TST and TIB were 2.7 minutes and 4.3 minutes respectively, the correlations were low. Timing of sleep onset and waketime were in better agreement and had a discrepancy of 36 and 8 minutes respectively. The limits of agreement on the Bland Altman plots were wide and affected by outliers (Fig 1). The largest variance was seen in sleep episodes that occurred during the day as seen on the plots for sleep onset and waketimes (Fig 1D and 1E).

**Table 2:** Comparison of manual sleep log entries to Fitbit data during 723 paired sleep episodes in 32 medical students and residents over 1 year of training between 2020 and 2021.

	Manual Sleep Log	Fitbit	Mean Comparison	Bland Altman	CCC^a^ (95% CI)
	Mean (SD)^b^	Mean (SD)^b^	Mean difference (SD)^b^	Limits of agreement	
Time in bed (minutes)	464.3 (107.7)	460.0 (92.9)	4.29 (127.2)	-250.16 to 258.76	0.20 (0.13–0.27)
Total sleep time (minutes)	435.6 (94.2)	432.9 (90.7)	2.74 (115.7)	-228.73 to 234.24	0.22 (0.15–0.29)
Sleep efficiency (%)	92.3% (9.5%)	94.7% (6.9%)	-2.4% (11.0%)	-24.40 to 19.60	0.12 (0.05–0.18)
Sleep onset time (24-hr clock)	16:26:43 (09:10:24)	15:50:11 (09:24:09)	36.5 (371.4)	-178.61 to 194.46	0.78 (0.74–0.80)
Wake-time (24-hr clock)	07:38:06 (03:07:00)	07:46:08 (03:05:18)	8.03 (139.0)	-264.32 to 280.39	0.72 (0.68–0.75)

^a^CCC = concordance correlation coefficient.

^b^SD = standard deviation.

### Study protocol compliance

Once all 50 participants received Fitbit, a total of 32 (64%) followed the instructions to synch the device to the cloud for de-identified data collection. Less than half (24, 48%) of participants completed the study in its entirety (i.e., entered all three rotations and the Exit Questionnaire) and only 15 (30%) wore the Fitbit device for the full 6 weeks. There was no difference in study completion in residents (52%) as compared to medical students (44%) or between rotation type.

The most common reason for not completing the study were: “My work schedule got in the way” (10, 67%), “It was too difficult to keep the sleep log” (9, 60%), “Forgot to wear my FitBit” (8, 53%), “I forgot to complete the surveys during the correct rotation” (5, 33%) and “Too many questions” (5, 33%). The estimated time to complete each data collection form was between 10–20 minutes for 61% of the participants and over 20 minutes for the remaining 39% of participants. Over half (53%) used the paper log to collect data daily and then enter it electronically at the end of the 2-week period (60%). Recurrent themes from the narrative feedback on the study design were that synching and wearing the Fitbit was too hard and the electronic format for data collection (i.e., REDCap survey system) took too much time. Others suggested creating a user-friendly smartphone application for data collection.

### Missingness analysis

**Fig 2:**
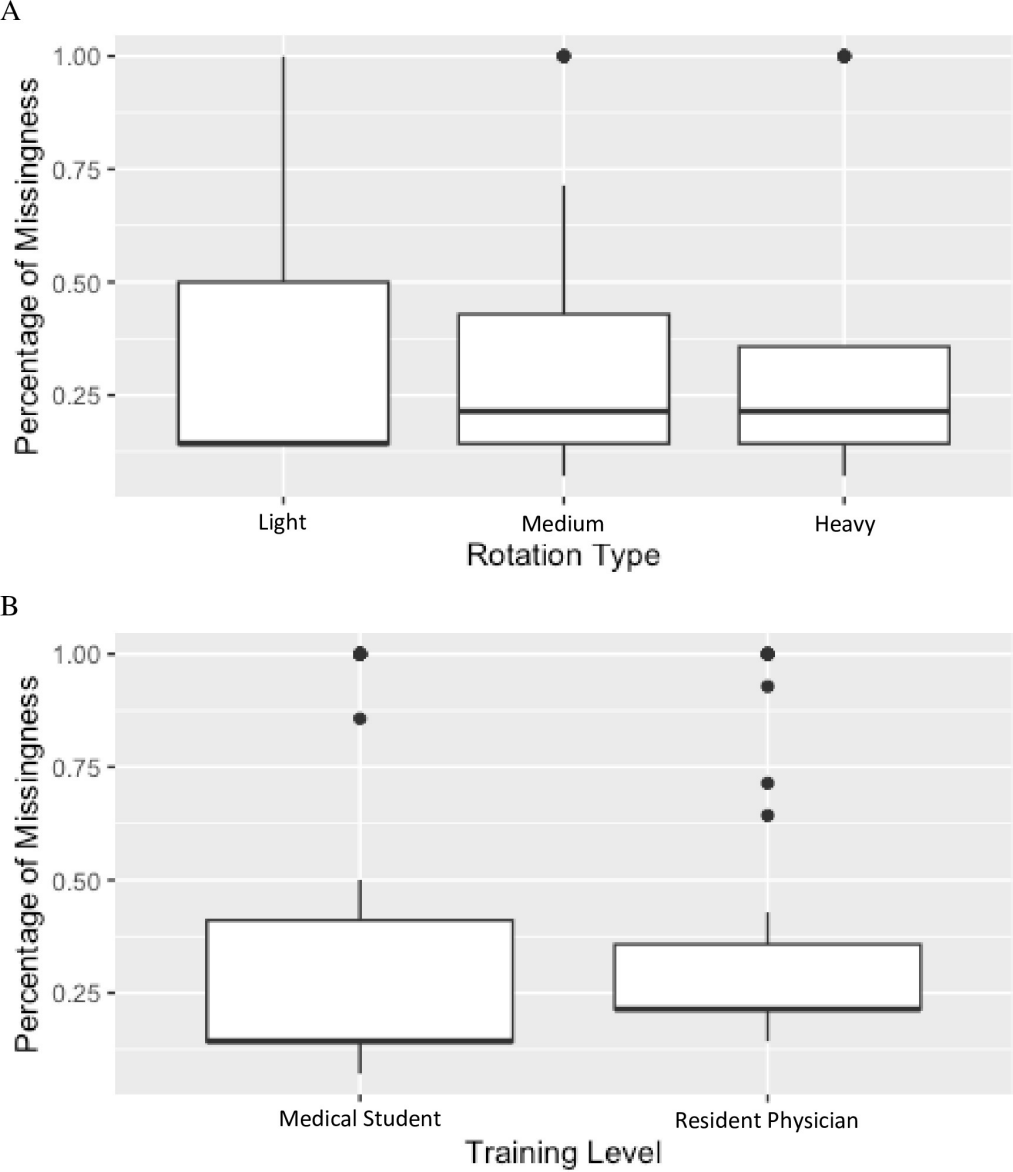
Percentage of data missing across rotation type and training level. Missingness of data collection of sleep patterns in 25 medical students and 25 residents during three different types of rotations: Light, medium and heavy clinical burden (A) and between the two training levels (B). The differences did not meet statistical significance.

There were no statistical differences in missingness of data between rotation type or medical students when compared to residents (Fig 2). When conducting additional analyses including sex, age, training level, working in your home area, insomnia, mood disorder and body mass index (BMI), the only variable associated with missing data was BMI.

### Differences between types of rotations and participants

Self-reported daily hours worked increased from the light to the medium and the heavy clinical rotations with a decline in total sleep time from 7.7 hours to 6.7 hours from the light to the heavy rotations (p<0.001). Sleep health scores, burnout, stress and quality of life scores worsened as the clinical burden increased. The proportions of participants who missed a life event, spent not enough time with family and who reported committing an error also increased with the heavier clinical rotations (Table 3).

**Table 3:** Comparisons between different clinical rotations in sleep patterns, stress and hours worked.

	*Light* clinical rotation (n = 30)	*Medium* clinical rotation (n = 28)	*Heavy* clinical rotation (n = 32)
Average daily hours worked	4.8 (1.5) ^**c**^	7.8 (2.1) ^**b**^	10.0 (2.0) ^**b**^ ^**c**^
Average daily hours slept	7.7 (0.64) ^**a**^ ^**c**^	7.1 (0.80)^**a**^	6.7 (0.88) ^**c**^
Days off in the 4-week rotation	4.8 (3.0) ^**a**^ ^**c**^	3.2 (2.5)^**a**^	3.1 (2.0) ^**c**^
Sleep health score	29.4 (3.7) ^**a**^ ^**c**^	26.4 (4.4)^**a**^	26.5 (4.5) ^**c**^
Perceived Stress Scale Score	20.4 (7.7) ^**a**^ ^**c**^	24.5 (7.3)^**a**^	26.0 (7.9) ^**c**^
Health related quality of life score	27.4 (3.7) ^**c**^	26.5 (3.4) ^**b**^	23.9 (5.3) ^**b**^ ^**c**^
Burnout scores	4.9 (3.4) ^**a**^ ^**c**^	6.7 (2.8)^**a**^	7.7 (3.5) ^**c**^
Missed a life event	3 (10%) ^**a**^ ^**c**^	12 (42.9%) ^**a**^ ^**b**^	25 (75.8%) ^**b**^ ^**c**^
Spent enough time with family and friends	25 (83.3%) ^**a**^ ^**c**^	10 (35.7%) ^**a**^ ^**b**^	1 (3%) ^**b**^ ^**c**^
Reported this was a difficult rotation for them	1 (3.3%) ^**a**^ ^**c**^	13 (46.4%) ^**a**^ ^**b**^	30 (90.9%) ^**b**^ ^**c**^
Committed a medical error	0 ^**c**^	4 (14.3%) 2 out of 4 due to lack of sleep	6 (19.4%) ^**c**^ 4 out of 6 due to lack of sleep
Screened positive for depression	5 (16.7%)	8 (28.6%)	12 (37.5%)
Correlations between burnout scores and sleep, work and quality of life			
**Sleep health score** and burnout score	r = -0.533^d^	r = -0.524^d^	r = -0.352^d^
Average **daily hours worked** and burnout score	r = -0.173	r = 0.004	r = 0.449^d^
Average **daily hours slept** and burnout score	r = -0.452^d^	r = -0.364	r = -0.436^d^
Health related **quality of life** and burnout score	r = -0.399^d^	r = -0.294	r = -0.614^d^

The participants who entered data on each rotation are shown below the rotation type as (n =) Data are presented as mean (SD) or n(proportion of column) unless otherwise specified. All comparisons performed using paired t-tests or Chi-square testing^a-c^.

^**a**^denotes p<0.05 when comparing the *light* rotation to the *medium* rotation.

^**b**^denotes p<0.05 when comparing the *medium* rotation to the *hard* rotation.

^**c**^denotes p<0.05 when comparing the *hard* rotation to the *light* rotation.

^d^Pearson’s correlations with p<0.05.

There were significant inverse correlations between burnout and sleep health scores, total number of hours slept per day, and quality of life in each type of clinical rotation (Table 3). Average daily hours worked was associated with burnout in heavy clinical rotation (r = 0.449, p = 0.013), but not in the other rotation-types. When adjusting for all of these variables, only sleep health score remained independently associated with burnout scores on the light rotation (β = -0.459, p = 0.004) and on the medium rotation (β = -0.581, p = 0.008).

## Discussion

This study aimed to assess the consistency of data provided by commercially available devices compared to manual sleep logs among physicians-in-training. Although the statistical correlations of the Fitbit TST, TIB, and SE% were found to be weak in our current study, the reported discrepancies for each individual measure may still be acceptable in practice. For instance, discrepancies of 2 minutes or a 2% difference in SE% may be tolerable. A review of literature reveals prior research that supports these findings. Studies employing Fitbit devices in general healthy adults indicates similar agreement between sleep duration reported in sleep logs and that derived from the Fitbit Inspire 2 [28] and Fitbit Flex [29] devices. In other studies of healthy young adults, Fitbit was able to estimate TIB, TST, SE%, sleep onset latency, and wake after sleep onset with minimal bias during the nocturnal sleep episodes [30] but mishandled daytime sleep episodes [31]. This aligns with our observation of low CCC values related to data variability from daytime sleep episodes.

In the studies investigating multiple dimensions of sleep health in physicians, few directly compare sleep log data to that from commercial wearable devices. Existing studies found in this area either rely on sleep logs alone, such as study of sleep banking in internal medicine residents by Cushman et al [32], or on Fitbit data alone, such as the study of 26 surgical residents wearing Fitbit devices on night float and home call [33]. To our knowledge, no study in physicians-in-training has directly compared sleep logs to commercial actigraphy device, which would allow for an evaluation of the external validity of our findings.

A consistent challenge in the literature regarding the sleep habits of physicians or physicians-in-training is the difficulty in obtaining comprehensive data. This challenge was evident in our study’s completion rate. Similarly, in the aforementioned Cushman study, only 25 of 50 eligible residents (50%) were able to complete a manual sleep log over the intended 11 days [32]. This issue is not unique in physicians; it is also seen in studies involving non-physicians. For instance, a Mayo clinic study of 84 hypersomnia patients evaluated by actigraphy reported that only 25 (30%) had complete capture of sleep log data alongside actigraphy use [34].

To inform future studies and contribute to the literature, it is important to note that the agreement between sleep logs and commercial actigraphy measurements met the recommended thresholds set by the American Academy of Sleep Medicine (AASM) for actigraphy to evaluate sleep disorders. For the AASM, the minimal allowable mean difference between clinical-grade actigraphy and sleep logs are within 2.5–5.0% for SE%, and 20 minutes of TST, sleep onset time and waketime [35]. While the difference in sleep onset time and the thresholds of the 95% CI of our data fell outside of the acceptable range per the AASM [35], this could be attributed to our small sample size and combining night time with daytime sleep episodes.

Our findings contribute to the growing body of evidence suggesting that studies on the sleep of physicians should minimize the data capture burden to reduce study attrition rates. The results that clinical rotation work load and training level did not correlate with ability to complete the study protocol provide guidance for future study design. Additionally, the finding that the only demographic variable associated with poorer study participation was BMI–a known risk factor for study attrition previously reported in the literature [36]–warrants further investigation in future research. Future studies should also carefully consider the duration of data collection. If the primary outcome is nightly TST, a sampling duration of 3 to 5 nights offers sufficient accuracy. However, for a comprehensive examination of monthly variations in habitual sleep, a more extensive period of 9 to 14 nights is recommended [37]. Based on our findings, a data collection of 9–10 days of wearing a commercial device to capture nighttime sleep measured in conjunction with a log of daytime sleep and shift-work times may be optimal for data collection and reliability in describing sleep patterns of physicians.

Given the exploratory nature of this pilot study, our initial intent did not include having a sample size adequate for detecting significant differences in sleep health and burnout across various rotations. Despite low compliance rates, we were still able to demonstrate that residents and medical students experience significant loss of sleep health and quality of life with an increase in stress and burnout as clinical duties increase. Our findings suggest that clinical rotation type is an important variable to include in future studies involving sleep in this population. This is consistent with a prospective observational trial of 26 surgical residents who wore Fitbit devices during home call, night float and in-house call. Those authors also found that changes in clinical burden detrimentally impacted physical activity and sleep hours [33]. Our results provide evidence that more work is needed in understanding how residents and medical students sleep in all clinical settings so we can ensure sustainability of this crucial workforce.

Several limitations must be acknowledged in this study. Firstly, poor protocol compliance rates hindered the attainment of robust sleep data. Secondly, the study was conducted at a single site, which limits the generalizability of the findings. Thirdly, the small sample size may have contributed to the substantial variability observed in the sleep data. Thus, further testing with a larger sample size across multiple sites is warranted to enhance the representativeness of the findings. Fourthly, self-reported information on sleep parameters may be susceptible to recall bias. However, the use of a sleep diary over 14 consecutive days likely mitigated this bias compared to single questions or recall-based scales. Fifthly, while Fitbit models have demonstrated reliability in determining sleep duration, pattern, and quality over consecutive nights under normal living conditions [38], caution is needed in interpreting results. Our study was conducted under unrestricted conditions in home settings, where various physical or environmental factors may influence uninterrupted sleep, especially in day and night shift working conditions. Additionally, our observations regarding daytime sleep suggest avenues for future research. Including larger and more diverse occupations of participants, particularly healthcare workers on regular night shifts, and tracking sleep over periods longer than two weeks could offer valuable insights but may be hampered by data capture concerns.

## Conclusion

Commercial wearable devices such as Fitbit provided acceptable estimates of total sleep time, efficiency, sleep onset time, and waketime in physicians-in-training. Wearing the device and completing a sleep log for 14-consecutive days simultaneously is not a pragmatic study design in this population. Daytime sleep episodes complicate analyses of physician sleep health. Future studies with streamlined sleep data collection are urgently needed as our evidence shows physicians-in-training have poor sleep health leading to burnout, stress, and a lower quality-of-life.
